# Supplementary material for: Violence against women migrant workers in Thailand: A cross-sectional study on experiences, impact, and help seeking
Source: PLoS One. 2026 Apr 1;21(4):e0345790. doi: 10.1371/journal.pone.0345790 (PMC13042863; doi:10.1371/journal.pone.0345790)
Supplement: Attachment — Submitted filename: Recommendations.pdf [file pone.0345790.s003.pdf]

Recommendations:

The manuscript presents original and policy-relevant empirical findings and predominantly satisfies PLOS ONE's scientific standards (robust analyses, ethical approval, and clarity of presentation). But before the paper can be accepted, some important problems need to be fixed: (1) inconsistent or unclear information about ethics approval, (2) incomplete or inconsistent information about data availability that doesn't follow PLOS policy, (3) not enough methodological detail in some areas (sampling, questionnaire validation, handling missing data, power/sample-size justification, and selection of covariates), and (4) clarification about how the findings overlap with those that have already been published. Below, I list the strengths, concerns (with proof from the file that was sent in), and specific changes that need to be made.

Strengths

- The subject matter is significant and insufficiently researched; violence against female migrant workers in Thailand is critically pertinent to policy, and the manuscript contributes empirical data from various provinces.
- The sample size is sufficiently large (n = 494) for cross-sectional prevalence and logistic regression analyses.
- The questionnaire seems to be based on WHO tools and has extra areas (economic abuse, cyberbullying) with an internal reliability score of about 0.8–0.9, which makes the measurement more credible.
- Analyses employ logistic regression, yielding both crude and adjusted odds ratios (AOR); the models account for age, education, and marital status, and display confidence intervals (CIs) and p-values. There are tables of AOR/COR that are easy to read and understand.

Major Concerns

1. **Ethics approval information that isn't always clear (needs to be fixed and made clear).**
    - There are two different COA numbers in the submission: COA.No.232/2020 and COA.No.196/2021. These numbers are shown in different parts of the file. The Methods section says that data collection was approved (COA.No.196/2021), but other administrative fields say COA.No.232/2020.

Please clarify which protocol(s) approved this study, the name of the committee that did so, the approval number(s) and date(s), and whether any changes or secondary analyses were included in the approval. If there are more than one approval, please explain clearly why (for example, if there are different substudies).

2. **The data availability statement does not follow PLOS ONE's rules as they are now written or make inconsistent claims.**

    - The manuscript makes the claim "All relevant data are within the manuscript and its Supporting Information files" in several places. It also says that "datasets used and analyzed are available from the corresponding author upon reasonable request," but qualitative transcripts are not available because they are sensitive and are held by a third party through UN Women. PLOS ONE generally wants the quantitative data that supports the findings to be publicly accessible in a repository or stored with the right protections. "Available on request" is usually not enough. Please:

        - Please say clearly which quantitative dataset(s) (like an anonymized survey CSV) will be deposited and give the repository name and accession/DOI (or say why deposition is not possible).

        - If you really can't share qualitative data, be clear about the exact limits and give people a way to get to it in a controlled way (for example, by giving them de-identified excerpts, a contact for the institutional data access committee, or a URL/email for the data custodian). The current contact information for UN Women is helpful, but it doesn't include a full compliance plan.

3. **There is some overlap with previous publications, so it needs to be made clear that it is new and not a duplicate.**

    - The manuscript states, "partial findings were previously published (Chuemchit et al., 2024) focusing on discrimination...." (Lines 127-128). The authors must explicitly delineate (a) the analyses/results/figures/tables that are novel in this submission, (b) any content that overlaps with the preceding paper, and (c) the rationale for why the current paper does not represent redundant publication. Give citations and, if you can, a short table of overlap (for example, "Table X in previous paper = data on discrimination; this paper uses the same sample but presents new analyses on lifetime IPV/N-IPV and help-seeking"). The journal policy says this is necessary.

4. **Sampling and generalizability: the limitations of snowball sampling require more thorough examination and the quantification of potential bias.**

    - Snowball sampling is suitable for elusive populations but is subject to selection bias. The manuscript recognizes its limitations; however, it should also delineate recruitment chains, seed selection criteria, the number of waves employed, any efforts made to diversify seeds, and offer a critical analysis of how these factors may skew prevalence estimates (e.g., through oversampling survivors). If possible, show sensitivity analyses or compare the demographics of the sample to external benchmarks (like national or regional distributions of migrant workers) to see how representative it is.

5. **Statistical methods: need more detail and checks for strength.**

    - The main methods used were logistic regressions and taking into account age, education, and marital status. Please give:

        - Why we chose these covariates instead of others, like income or length of stay, which are shown in tables as exposures.
        - Handling of missing data (how much data is missing for each variable and whether listwise deletion, imputation, or other methods were used).
        - If the correction for multiple comparisons was taken into account (a lot of associations were tested).
        - If you can, put unadjusted and fully adjusted model tables in the main text or the supplement.

6. **There is no sample size or power calculation.**

    - The paper must indicate whether a priori sample size or power calculations were conducted to substantiate the use of n=494 for the primary outcomes (prevalence estimates, detection of associations). If none was done, add a short discussion of detectable effect sizes based on the sample.

7. **Measurement validation: additional information required regarding questionnaire validation and reliability.**

    - The manuscript states that three experts verified content validity and presents reliability coefficients (α = 0.8, 0.9). Please include information on how experts were chosen, whether cognitive interviewing or pilot testing was

done, how translation and back-translation were done, and item-level psychometrics (if available). This is significant as the instrument was adapted from WHO tools to incorporate new domains (economic/cyber violence).

8. Interpretation of the findings.

- The Discussion section presently contains multiple assertions that suggest causality (e.g., “X leads to Y,” “Factor A causes higher rates of violence,” “B results in increased risk of C”). Because this is a cross-sectional study, the data cannot be used to draw conclusions about cause and effect. Associations can be reported, but establishing cause-and-effect relationships necessitates longitudinal or experimental designs.
- The authors need to change the language in the Discussion to show that the findings are related. For instance, phrases like "X is linked to Y" or "people who said X were more likely to say Y" would be better than causal statements.
- The study makes a number of cross-country comparisons, which suggest that the patterns or prevalence found in this study are "higher" or "lower" than those found in other contexts. It is important to be careful when making these comparative claims because the sampling frames, measurement tools, and study designs are all very different. For instance, it would be more accurate to say, "The prevalence observed in this non-probability sample of migrant women appears higher than figures reported in national surveys from country Z; however, differences in methodology limit direct comparability."
