# Supplementary material for: Violence against women migrant workers in Thailand: A cross-sectional study on experiences, impact, and help seeking
Source: PLoS One. 2026 Apr 1;21(4):e0345790. doi: 10.1371/journal.pone.0345790 (PMC13042863; doi:10.1371/journal.pone.0345790)
Supplement: Attachment — Submitted filename: Reviewers feedback.docx [file pone.0345790.s002.docx]

**Reviewer’s Feedback**

The manuscript titled *“Violence Against Women Migrant Workers in Thailand: A Cross-Sectional Study on Experiences, Impact, and Help Seeking”* presents an important and timely contribution to the field of public health and gender-based violence research. The topic is highly relevant, particularly in the Southeast Asian context where migrant women face intersecting vulnerabilities due to gender, socioeconomic status, and migration status. The study is ethically sound, methodologically rigorous, and presented in a clear and structured manner. Below are detailed comments and suggestions:

**1. Technical Soundness and Data Support for Conclusions**

The research is methodologically solid. The use of a cross-sectional design is appropriate for assessing the prevalence and correlates of violence among women migrant workers. The sample size (n = 494) across seven provinces provides substantial breadth for descriptive and inferential analysis.
The study instruments are well-grounded in existing validated tools, particularly the WHO multi-country study questionnaire, which enhances validity. Internal reliability is strong (Cronbach’s α = 0.8–0.9).

The conclusions are appropriately drawn and align with the results. The authors correctly refrain from making causal claims and discuss limitations related to the sampling strategy and potential biases. The policy implications and recommendations are relevant and well-articulated.

**Suggestion:** Consider briefly emphasizing how findings can inform specific interventions or existing national policies addressing migrant women’s welfare in Thailand to enhance applied significance.

**2. Statistical Analysis**

The statistical analyses are appropriate and performed with good rigor. Descriptive statistics and logistic regression analyses (both crude and adjusted models) are used effectively to explore associations between demographic and contextual variables with IPV and N-IPV. Adjustments for potential confounders (age, education, marital status) are suitable.
The tables are clear, and significant results are correctly interpreted in the discussion.

**Minor recommendations:**

- State whether multicollinearity among predictors was tested and ruled out.
  These additions would further strengthen transparency and confidence in the analytical rigor.

**3. Data Availability**

The **Data Availability Statement** is transparent and ethically justified. Quantitative data are reported to be fully available within the manuscript, while qualitative data are restricted due to the sensitive nature of violence narratives. This is an acceptable limitation under ethical standards; however, it does not fully comply with PLOS ONE’s open data policy. However, the restricted access to qualitative data is understandable, provided that ethical constraints are clearly documented, as already done.

**4. Ethical Considerations**

The study adheres to appropriate ethical standards, with approval from Chulalongkorn University Ethics Review Committee (COA.No.196/2021). Informed consent procedures were well described, and participants’ confidentiality was prioritized.
The study deals with a highly sensitive topic and demonstrates commendable ethical awareness, especially in using trained female enumerators from migrant communities and providing referral information to participants after interviews.

**Overall Assessment**

This manuscript makes a **valuable empirical and policy-relevant contribution** to the understanding of violence against women migrant workers in Thailand. The design, analysis, and interpretation are appropriate, and the findings are clearly presented and well-supported by the data.

**Strengths:**

- Robust methodology and sample size
- Ethical and culturally sensitive data collection
- Clear presentation and actionable recommendations

**Areas for minor improvement:**

- State whether multicollinearity among predictors was tested and ruled out
- Consider briefly highlighting how the findings can inform specific interventions or align with existing national policies
