# Supplementary material for: Violence against women migrant workers in Thailand: A cross-sectional study on experiences, impact, and help seeking
Source: PLoS One. 2026 Apr 1;21(4):e0345790. doi: 10.1371/journal.pone.0345790 (PMC13042863; doi:10.1371/journal.pone.0345790)
Supplement: Attachment — Submitted filename: Response To Reviewers_MC.docx [file pone.0345790.s005.docx]

**Response of authors to comments from editors and reviewers on the manuscript**

**“Violence Against Women Migrant Workers in Thailand: A Cross-Sectional Study on Experiences, Impact, and Help Seeking”**

**Journal Requirements:**

When submitting your revision, we need you to address these additional requirements.

1. Please ensure that your manuscript meets PLOS ONE's style requirements, including those for file naming. The PLOS ONE style templates can be found at

<https://journals.plos.org/plosone/s/file?id=wjVg/PLOSOne_formatting_sample_main_body.pdf> and

[https://journals.plos.org/plosone/s/file?id=ba62/PLOSOne_formatting_sample_title_authors_affiliations.pdf](https://track.editorialmanager.com/CL0/https:%2F%2Fjournals.plos.org%2Fplosone%2Fs%2Ffile%3Fid=ba62%2FPLOSOne_formatting_sample_title_authors_affiliations.pdf/1/010f019b34d86229-a0ef328a-7f9c-4609-85cf-669a71e53d0c-000000/toLiUbaM36bQ04FzUmn_ZWvaPKIwu_EDxGd8IMFteM4=242)

***The authors have ensured that manuscript meets PLOS ONE's style requirements, including those for file naming.***

2. We note that only one reference number for your ethics documents is listed in the ethics section of your manuscript. Please add the original ethics document reference number (COA.No.232/2020) to your manuscript.

***Thank you for this important comment. The authors made a revision to add the original ethics document reference number (COA.No.232/2020) to the manuscript.***

3. We note that the grant information you provided in the ‘Funding Information’ and ‘Financial Disclosure’ sections do not match. When you resubmit, please ensure that you provide the correct grant numbers for the awards you received for your study in the ‘Funding Information’ section.

***Thank you for your comment. We have corrected the grant information and ensured that the correct and consistent grant numbers are now accurately reported in both the Funding Information and Financial Disclosure sections in the revised submission.***

4. Thank you for stating the following financial disclosure:

“This study was funded by the EU funded Spotlight initiative, Safe and Fair Program, UN Women Thailand (PSA-ROAP-2021-008).” Please state what role the funders took in the study. If the funders had no role, please state: "The funders had no role in study design, data collection and analysis, decision to publish, or preparation of the manuscript." If this statement is not correct you must amend it as needed. Please include this amended Role of Funder statement in your cover letter; we will change the online submission form on your behalf.

***Thank you for this instruction. In the manuscript document and cover letter, the authors have added the information as “The funders had no role in data collection and analysis, decision to publish, or preparation of the manuscript.”***

5. For studies involving third-party data, we encourage authors to share any data specific to their analyses that they can legally distribute. PLOS recognizes, however, that authors may be using third-party data they do not have the rights to share. When third-party data cannot be publicly shared, authors must provide all information necessary for interested researchers to apply to gain access to the data. (<https://journals.plos.org/plosone/s/data-availability#loc-acceptable-data-access-restrictions>) For any third-party data that the authors cannot legally distribute, they should include the following information in their Data Availability Statement upon submission:

1) A description of the data set and the third-party source

2) If applicable, verification of permission to use the data set

3) Confirmation of whether the authors received any special privileges in accessing the data that other researchers would not have

4) All necessary contact information others would need to apply to gain access to the data

***Thank you for the comments. This manuscript uses the data of our own project. There is no third-party data that is used for data analysis in this paper.***

6. If the reviewer's comments include a recommendation to cite specific previously published works, please review and evaluate these publications to determine whether they are relevant and should be cited. There is no requirement to cite these works unless the editor has indicated otherwise.

***The authors are well noted and thank you for this instruction. We have followed this instruction in the revised manuscript.***

**Comments to the Author**

1. Is the manuscript technically sound, and do the data support the conclusions?

- Reviewer #1: Yes
- Reviewer #2: Yes

2. Has the statistical analysis been performed appropriately and rigorously?

- Reviewer #1: Yes
- Reviewer #2: Yes

3. Have the authors made all data underlying the findings in their manuscript fully available? In addition to summary statistics, the data points behind means, medians and variance measures should be available. If there are restrictions on publicly sharing data—e.g. participant privacy or use of data from a third party—those must be specified.

- Reviewer #1: No
- Reviewer #2: No

***Thank you for your comments. The authors have revised the “Data availability statement” in the manuscript as “All relevant data are within the paper. Quantitative data is openly accessible.” We have uploaded the dataset to the system. The qualitative data are totally not presented/ described/ used/ analysed in this paper. Therefore, the qualitative data is not relevant to mention for this current paper.***

**Comments from reviewers**

**Reviewer #1:**

The manuscript titled *“Violence Against Women Migrant Workers in Thailand: A Cross-Sectional Study on Experiences, Impact, and Help Seeking”* presents an important and timely contribution to the field of public health and gender-based violence research. The topic is highly relevant, particularly in the Southeast Asian context where migrant women face intersecting vulnerabilities due to gender, socioeconomic status, and migration status. The study is ethically sound, methodologically rigorous, and presented in a clear and structured manner. Below are detailed comments and suggestions:

***Thank you for your appreciation and support for our manuscript. The authors really appreciate your comments and suggestions.***

**1. Technical Soundness and Data Support for Conclusions**

The research is methodologically solid. The use of a cross-sectional design is appropriate for assessing the prevalence and correlates of violence among women migrant workers. The sample size (n = 494) across seven provinces provides substantial breadth for descriptive and inferential analysis. The study instruments are well-grounded in existing validated tools, particularly the WHO multi-country study questionnaire, which enhances validity. Internal reliability is strong (Cronbach’s α = 0.8–0.9). The conclusions are appropriately drawn and align with the results. The authors correctly refrain from making causal claims and discuss limitations related to the sampling strategy and potential biases. The policy implications and recommendations are relevant and well-articulated.

**Suggestion:** Consider briefly emphasizing how findings can inform specific interventions or existing national policies addressing migrant women’s welfare in Thailand to enhance applied significance.

***Thank you for your comments. The authors have revised the manuscript according to the reviewer’s suggestions as follows.***

*Conclusion of the Abstract (line 53 – 59): “Key strategies include strengthening workplace-based protections, multilingual reporting mechanisms, peer networks, culturally relevant education, and individual- and community-level interventions within labour, health, and social protection systems. Collaborative efforts among policymakers and stakeholders are vital for the prevention of and response to violence and for ensuring the safety of women migrant workers.”*

*Recommendations and conclusions (Line 472 – 503): “Our study identified a high prevalence of multiple forms of violence against women migrant workers, highlighting an urgent need for targeted, survivor-centred actions that address key risk factors identified in this study, including area of residence, duration of stay, monthly income, perceived safety, Thai language proficiency, and job or income loss during the COVID-19 pandemic. Policymakers and service providers ..., including strengthening multilingual and migrant-sensitive services within existing mechanisms such as workplace-based protections, labour inspection systems, Migrant Worker Assistance Centres, and health-sector One-Stop Crisis Centres in Thailand. Expanding Thai language programs and communication support is essential to improve migrant women’s ability to understand their rights, report violence, and access appropriate formal support. In addition, ... complementing formal support systems that are currently underutilized. ... and aligned with national gender equality, anti-trafficking, and business and human rights frameworks to promote violence prevention in migrant communities and workplaces. Furthermore, ... supporting evidence-based implementation and evaluation of national policies addressing migrant women’s welfare. ... In summary, ... while reinforcing Thailand’s existing legal and policy commitments to violence prevention, gender equality, and migrant worker protection. Importantly, ... ensuring that national policies are translated into practical, survivor-centred actions that contribute to the elimination of violence against this marginalized population.”*

**2. Statistical Analysis**

The statistical analyses are appropriate and performed with good rigor. Descriptive statistics and logistic regression analyses (both crude and adjusted models) are used effectively to explore associations between demographic and contextual variables with IPV and N-IPV. Adjustments for potential confounders (age, education, marital status) are suitable.
The tables are clear, and significant results are correctly interpreted in the discussion.

**Minor recommendations:** State whether multicollinearity among predictors was tested and ruled out. These additions would further strengthen transparency and confidence in the analytical rigor.

***Thank you for your comments. The authors have revised the manuscript according to the reviewer’s suggestions as follows.***

*Line 248 – 249: “Multicollinearity among predictors was tested; Variance Inflation Factor (VIF) of all variables were ≤ 5, indicating no issue of multicollinearity.”*

**3. Data Availability**

The **Data Availability Statement** is transparent and ethically justified. Quantitative data are reported to be fully available within the manuscript, while qualitative data are restricted due to the sensitive nature of violence narratives. This is an acceptable limitation under ethical standards; however, it does not fully comply with PLOS ONE’s open data policy. However, the restricted access to qualitative data is understandable, provided that ethical constraints are clearly documented, as already done.

***Thank you for your comments. The authors have revised the “Data availability statement” in the manuscript as “All relevant data are within the paper. Quantitative data is openly accessible.” We have uploaded the dataset to the system. The qualitative data are not presented/ described/used/ analysed in this paper. Therefore, the qualitative data is not relevant to mention for this current paper.***

**4. Ethical Considerations**

The study adheres to appropriate ethical standards, with approval from Chulalongkorn University Ethics Review Committee (COA.No.196/2021). Informed consent procedures were well described, and participants’ confidentiality was prioritized. The study deals with a highly sensitive topic and demonstrates commendable ethical awareness, especially in using trained female enumerators from migrant communities and providing referral information to participants after interviews.

***The authors express sincere thanks to the reviewer. The original ethics approval for this study was granted by the Chulalongkorn University Ethics Review Committee (COA.No.232/2020). The approval number COA.No.196/2021 refers to the approved extension. We have revised the manuscript to clearly report both ethics reference numbers.***

**Overall Assessment**

This manuscript makes a **valuable empirical and policy-relevant contribution** to the understanding of violence against women migrant workers in Thailand. The design, analysis, and interpretation are appropriate, and the findings are clearly presented and well-supported by the data.

**Strengths:**

- Robust methodology and sample size
- Ethical and culturally sensitive data collection
- Clear presentation and actionable recommendations

**Areas for minor improvement:**

- State whether multicollinearity among predictors was tested and ruled out
- Consider briefly highlighting how the findings can inform specific interventions or align with existing national policies

***Thank you for your comments and suggestions. The authors have revised the manuscript according to the reviewer’s recommendations as mentioned above.***

**Reviewer #2**

The manuscript presents original and policy-relevant empirical findings and predominantly satisfies PLOS ONE’s scientific standards (robust analyses, ethical approval, and clarity of presentation). However, before the paper can be accepted, some important problems need to be fixed: (1) inconsistent or unclear information about ethics approval, (2) incomplete or inconsistent information about data availability that does not follow PLOS policy, (3) insufficient methodological detail in some areas (sampling, questionnaire validation, handling missing data, power/sample-size justification, and selection of covariates), and (4) clarification regarding how the findings overlap with those that have already been published.

Below, I list the strengths, concerns (with proof from the file that was sent in), and specific changes that need to be made.

***The authors appreciate the reviewer’s comments and suggestions. We have made the required revision to the manuscript as detailed below.***

**Strengths**

• The subject matter is significant and insufficiently researched; violence against female migrant workers in Thailand is critically pertinent to policy, and the manuscript contributes empirical data from various provinces.

• The sample size is sufficiently large (n = 494) for cross-sectional prevalence and logistic regression analyses.

• The questionnaire appears to be based on WHO tools and includes additional domains (economic abuse, cyberbullying), with internal reliability scores of approximately 0.8–0.9, which strengthens measurement credibility.

• Analyses employ logistic regression, yielding both crude and adjusted odds ratios (AOR). The models account for age, education, and marital status, and report confidence intervals (CIs) and p-values. The AOR/COR tables are clear and easy to interpret.

***The authors appreciate the reviewer’s comments and suggestions. We have made the required revision to the manuscript as detailed below.***

**Major Concerns**

**1. Ethics approval information is unclear and inconsistent (must be clarified).**

• Two different COA numbers are reported in the submission: COA.No.232/2020 and COA.No.196/2021. These numbers appear in different sections of the file. The Methods section states that data collection was approved under COA.No.196/2021, while other administrative fields report COA.No.232/2020.

Please clarify which protocol(s) approved this study, the name of the approving committee, the approval number(s) and date(s), and whether any amendments or secondary analyses were included in the approval. If more than one approval was obtained, please clearly explain why (for example, if different sub-studies were involved).

***Thank you for your comments. This study was approved by the Chulalongkorn University Ethics Review Committee (COA.No.232/2020), Thailand, on 8 October 2020, with an approved extension under COA.No.196/2021 on 8 October 2021. We have revised the manuscript to clearly report both ethics reference numbers. There were no amendments and no secondary analyses made in this current paper as well as in the original research project.***

**2. The data availability statement does not comply with current PLOS ONE policy and contains inconsistencies.**

• The manuscript repeatedly states that “All relevant data are within the manuscript and its Supporting Information files.” It also claims that “datasets used and analysed are available from the corresponding author upon reasonable request,” while noting that qualitative transcripts are unavailable because they are sensitive and held by a third party (UN Women).

PLOS ONE generally requires that quantitative data supporting the findings be publicly accessible via a repository or shared under appropriate protections. “Available upon request” is typically insufficient. Please address the following:

o Clearly specify which quantitative dataset(s) (e.g., an anonymized survey CSV) will be deposited, including the repository name and accession number/DOI, or explicitly explain why deposition is not possible.

o If qualitative data cannot be shared, clearly state the limitations and provide a controlled access mechanism (e.g., de-identified excerpts, contact details for an institutional data access committee, or a URL/email for the data custodian). While the current UN Women contact information is useful, it does not yet constitute a full compliance plan.

***Thank you for your comments. The authors have revised the “Data availability statement” in the manuscript as “All relevant data are within the paper. Quantitative data is openly accessible.” We have uploaded the dataset to the system. The qualitative data are not presented/ described/used/ analysed in this paper. Therefore, the qualitative data is not relevant to mention for this current paper.***

**3. Overlap with previous publications must be clearly delineated to avoid redundancy.**

• The manuscript states that “partial findings were previously published (Chuemchit et al., 2024) focusing on discrimination…” (Lines 127–128).

The authors must explicitly describe:

(a) which analyses, results, figures, and tables are novel in this submission;

(b) which elements overlap with the prior publication; and

(c) why the current manuscript does not constitute redundant publication.

Please include appropriate citations and, if possible, a brief table summarizing overlap (e.g., “Table X in the previous paper reports discrimination data; the current paper uses the same sample but presents new analyses on lifetime IPV/N-IPV and help-seeking”). This is required under journal policy.

***Thank you for this important comment. The authors have put the required information in the revised manuscript according to your suggestions as below.***

Materials and methods line 137 – 145. “*Although this study uses the same sample from the research project and therefore shares general characteristics with the preceding paper, the analyses and results presented in the current paper, including all tables and figures, are entirely novel. This paper presents new analyses and critical findings on women’s lifetime experiences of IPV and non-IPV, their impacts, associated factors, and patterns of help-seeking, including ways help was received, knowledge of help-seeking, and future help-seeking plans. Accordingly, the current paper addresses separate analytical aims and contributes to original evidence that is not reported in the preceding publication and therefore does not constitute redundant publication. Findings of this paper provide a significant contribution to understanding the multifaceted challenges faced by migrant women workers.”*

**4. Sampling and generalizability require deeper examination and bias assessment.**

• Snowball sampling is appropriate for hard-to-reach populations but is subject to selection bias. While the manuscript acknowledges this limitation, it should also describe recruitment chains, seed selection criteria, the number of recruitment waves, and any steps taken to diversify seeds.

A more critical discussion is needed regarding how these factors may bias prevalence estimates (e.g., oversampling survivors). If feasible, include sensitivity analyses or comparisons between sample demographics and external benchmarks (such as national or regional migrant worker statistics) to assess representativeness.

***The authors thank to the reviewer for these important suggestions. Accordingly, revision was done in Study design and sampling techniques Line 166 – 176 and in discussion (limitation Line 463 – 465).***

*Seed selection criteria*

*“Initial seeds were purposively selected through community-based organizations and outreach workers based on eligibility, willingness to participate, and diversity in nationality, age, and employment sector.”*

*Efforts to diversify seeds*

*“To enhance diversity in recruitment chains, multiple seeds were initiated across different geographic locations, workplace settings, and migrant communities.”*

*Recruitment chains*

*“Recruitment proceeded through participant referral chains, with each participant invited to refer additional eligible participants from their social networks.”*

*Number of waves employed*

*“Recruitment continued for approximately five to ten referral waves, until the target sample size was achieved and no substantial new recruitment networks emerged.”*

*Comparison with external benchmarks*

*“The demographic characteristics of the sample, including age, nationality, and employment sector, were compared with available national and regional statistics on migrant workers in Thailand to assess the degree of representativeness.”*

*(limitation) Potential skewing of prevalence estimates*

*“Given the reliance on social networks, participants with prior experiences of violence or stronger community connections may have been more likely to be recruited, potentially leading to an overrepresentation of survivors and inflation of prevalence estimates.”*

**5. Statistical methods require additional detail and robustness checks.**

• Logistic regression models adjust for age, education, and marital status. Please clarify:

o The rationale for selecting these covariates instead of others (e.g., income or length of stay), which are presented as exposures in tables.

o How missing data were handled, including the extent of missingness for each variable and whether listwise deletion, imputation, or alternative methods were used.

o Whether any correction for multiple comparisons was applied, given the large number of associations tested.

o If possible, include both unadjusted and fully adjusted model results in the main text or supplementary materials.

***Thank you for this comment. The authors have put the required information in the revised manuscript according to your suggestions as below.***

*Statistical analysis Line 242 – 248: “Age, education, and marital status were selected as adjustment variables based on prior literature and conceptual relevance, as they are potentially associated with both exposure variables and violence outcomes. Other variables such as income and length of stay were not included as covariates because they were conceptually defined as primary exposure variables rather than confounders. Adjusting for these variables could constitute over adjustment and potentially obscure the associations of interest.”*

*Handling of missing data: There was no missing data for the variables included in the regression analyses. Therefore, all models were estimated using complete-case data, and no imputation or other missing data techniques were required.*

*Multiple comparisons: Given the exploratory nature of the analyses and the predefined theoretical framework guiding variable selection, no formal correction for multiple comparisons was applied. We interpreted the findings with caution, with emphasis placed on the magnitude, direction, and consistency of associations rather than on isolated statistically significant results.*

*Presentation of unadjusted and adjusted models: Unadjusted associations are presented in the main manuscript as crude odds ratios derived from simple logistic regression models. Adjusted analyses were conducted by controlling age, education, and marital status, which were identified a priori as key confounding variables and were applied consistently across all exposure models. Fully adjusted models including all measured variables were not performed or presented, as the exposure variables were conceptually defined as primary exposure variables rather than confounders. Simultaneous adjustment for these variables could result in over adjustment and compromise the interpretability of the associations of interest.*

**6. No sample size or power calculation is provided.**

• The manuscript should indicate whether an a priori sample size or power calculation was conducted to justify the use of n = 494 for the primary outcomes (prevalence estimates and association detection). If no such calculation was performed, please include a brief discussion of detectable effect sizes given the sample.

***Thank you for insightful comments. The authors have put the required information in the revised manuscript according to your recommendations as below.***

Study design and sampling techniques Line 156 – 165: “*The sample size was determined by feasibility considerations and recruitment capacity, given the hard-to-reach nature of migrant women workers and the use of snowball sampling. With a total sample of 494 participants, the study had adequate precision to estimate prevalence for common outcomes and sufficient power to detect associations of moderate magnitude in logistic regression analyses. Assuming a two-sided alpha level of 0.05 and outcome prevalence between 20% and 50%, this sample size would allow detection of odds ratios in the moderate range for exposures with reasonable prevalence in the sample. No formal a priori sample size or power calculation was conducted; therefore, smaller effect sizes or associations involving rare exposures may not have been detectable, and nonsignificant findings should be interpreted with caution.*”

**7. Measurement validation requires additional detail.**

• The manuscript reports that three experts assessed content validity and provides reliability coefficients (α = 0.8–0.9). Please add information on expert selection, whether cognitive interviews or pilot testing were conducted, the translation and back-translation process, and item-level psychometric properties (if available). This is especially important because the instrument adapts WHO tools and adds new domains (economic and cyber violence).

***Thank you for this comment. The authors have put the required information in the revised manuscript according to your suggestions as below.***

Research Instrument Line 191 – 200: “*The survey instrument was adapted with additional items developed to capture emerging forms of violence, including economic and cyber violence. Content validity was assessed by three purposively selected experts with expertise in gender-based violence, migrant health, and public health research, who reviewed the instrument for relevance, clarity, cultural appropriateness, and construct coverage. The questionnaire was translated using a forward–backward translation process conducted by bilingual translators, with discrepancies resolved by the research team to ensure conceptual equivalence. The instrument was pretested with participants from the target population to assess clarity, comprehension, and flow, and minor revisions were made accordingly.*”

**8. Interpretation of findings requires more cautious language.**

• The Discussion section includes statements implying causality (e.g., “X leads to Y,” “Factor A causes higher rates of violence,” “B results in increased risk of C”). As this is a cross-sectional study, causal conclusions cannot be drawn. Associations may be reported, but causal inference requires longitudinal or experimental designs.

• Please revise the Discussion to use associative language (e.g., “X is associated with Y” or “participants reporting X were more likely to report Y”).

• The manuscript also makes cross-country comparisons suggesting higher or lower prevalence than in other contexts. Such comparisons should be framed cautiously, as sampling frames, measurement tools, and study designs differ substantially. For example, it would be more accurate to state:

“The prevalence observed in this non-probability sample of migrant women appears higher than figures reported in national surveys from country Z; however, methodological differences limit direct comparability.”

***Thank you for insightful comments. The authors have made the required revision in the discussion section of revised manuscript according to your recommendations as below.***

*Women residing in provinces other than Bangkok were associated with lower odds of reporting both IPV and N-IPV (line 412 – 413)*

*women with a higher income in this study were associated with lower odds of reporting violence experiences (Line 425 – 426)*

*women with a longer duration of stay in Thailand and women who perceived themselves to be unsafe in Thailand were associated with an increased likelihood of IPV and N-IPV. (line 437 – 438)*

*women who lost their jobs during the COVID-19 pandemic were associated with higher likelihood of reporting IPV and N-IPV experiences (Line 450 – 451)*

*Line 363 – 366: This prevalence is higher than that of violence against the general population of women in Myanmar, Laos, and Cambodia according to each country’s nationwide surveys [25-27]; however, differences in methodology limit direct comparability.*
